# Extracellular Vesicles as Emerging Tools in Canine Mammary Tumors: Potential Applications and Perspectives

**DOI:** 10.3390/ani16162478

**Published:** 2026-08-10

**Authors:** Luadna dos Santos e Silva, Debora Aparecida Pires de Campos Zuccari, Guilherme Henrique Tamarindo, Cintia Oliveira, Caroline Anacleto Rinaldi, Luiz Gustavo de Almeida Chuffa

**Affiliations:** 1 Laboratory of Molecular Cancer Research (LIMC), Faculty of Medicine of São José do Rio Preto (FAMERP), Avenida Brigadeiro Faria Lima, São José do Rio Preto 15090-000, Brazil; luadna.silva@edu.famerp.br; 2 Brazilian National Biosciences Laboratory (LNBio), Brazilian Center for Research in Energy and Materials (CNPEM), Rua Giuseppe Máximo Scolfaro, Campinas 13083-970, Brazil; guilherme.tamarindo@lnbio.cnpem.br; 3 Department of Anatomy, Institute of Biosciences, São Paulo State University (UNESP), Botucatu 18618-689, Brazil; cintia.oliveira26@unesp.br (C.O.); caroline.rinaldi@unesp.br (C.A.R.); luiz-gustavo.chuffa@unesp.br (L.G.d.A.C.)

**Keywords:** mammary tumors, dogs, extracellular vesicles

## Abstract

Breast cancer is one of the most prevalent malignancies in women worldwide and the most common malignant tumor in unspayed female dogs, highlighting the comparative relevance of this disease across these species. The release of EVs is increased in mammary tumors compared to normal tissue, with the quantity generally correlating with the tumor’s histological grade and aggressiveness. EVs carry a varied molecular load, mainly composed of proteins, RNAs, including microRNAs (miRNAs), lipids, and nucleic acids. These contents reflect the cell of origin and are related to the tumor profile (adenoma, adenocarcinoma, or carcinoma). EVs influence biological processes such as cell proliferation, migration, and invasion, as well as interfere with the immune response, potentially favoring tumor growth, disease progression, and metastasis formation, since they act as intercellular messengers, modulating the tumor microenvironment. Despite substantial advances in research related to EVs in human breast cancer, there is still a significant lack of studies focused on veterinary oncology, especially regarding the association of EVs with CMTs. In this context, this review aimed to compile the evidence available to highlight the importance of EVs in CMTs in female dogs, especially as promising tools for innovative strategies in veterinary medicine.

## Simple Summary

Knowledge regarding how extracellular vesicles (EVs) directly contribute to tumorigenesis, progression, invasiveness, and interaction with the tumor microenvironment in canine mammary tumors (CMTs) remains limited. In this context, although recent studies demonstrate the potential of these structures as biomarkers and mediators of tumor communication, significant gaps persist regarding clinical validation and, above all, the mechanistic understanding of their biological role. This review not only compiles available evidence but also highlights the need for integrative and functional approaches capable of elucidating the mechanisms underlying EV activity, thereby guiding the development of future studies with greater translational potential in veterinary oncology.

## 1. Introduction

### 1.1. Mammary Tumors in Female Dogs

Mammary tumors represent the second most commonly diagnosed type of cancer in female dogs, constituting a significant health problem in veterinary clinics, especially in unspayed animals [1]. Consistently, approximately 50% of CMTs exhibit malignant behavior, reinforcing the need for more precise diagnostic and prognostic strategies [2].

Despite the clinical relevance of these tumors, there is still limited information on the effectiveness of adjuvant systemic therapies, such as chemotherapy and radiotherapy, in the treatment of CMTs, which may be related, in part, to the high cost and limited availability of these procedures in veterinary practice. Although there are case reports and specific studies describing responses to chemotherapeutic agents such as doxorubicin, mitoxantrone, paclitaxel, and carboplatin, it remains uncertain whether chemotherapy can promote consistent benefit in terms of overall survival in female dogs with mammary tumors [3]. In this context, there are studies in the literature that focus on the discovery of new markers of the disease and its stage of progression.

### 1.2. Extracellular Vesicles

Yuan et al. [4] demonstrated that the AGR2 protein found in EVs from CMT cell lines participates in an unconventional secretion mechanism, dependent on endoplasmic reticulum stress and autophagy, promoting the release of proteins such as 14-3-3ε and α-actinin-4 in CMT cells. According to this study, these secreted factors act in cellular chemotaxis, suggesting an important role in tumor migration and potential invasiveness. The results indicate that pathways associated with non-classical secretion may contribute to cancer progression, highlighting AGR2 as a potential therapeutic target and as a regulator of tumor communication in the microenvironment. Investigations involving EVs have been progressively increasing, with reports of isolation and characterization of these structures from the plasma of various species, including dogs, highlighting the potential of these vesicles as promising tools for biomarker discovery [5]. In this context, the investigation of circulating biomarkers and tumor communication mechanisms mediated by EVs becomes particularly relevant, since these structures play a central role in modulating the tumor microenvironment, metastatic dissemination, and resistance to antineoplastic therapies. In addition to their diagnostic and prognostic potential, EVs emerge as promising therapeutic tools, either for their ability to transport bioactive molecules involved in tumor progression or for acting as natural platforms for the development of targeted drug delivery strategies [6,7,8].

EVs constitute a heterogeneous family of particles originating from both endosomal compartments and the plasma membrane. Initially considered merely byproducts of cellular metabolism, EVs are now recognized as central components of intercellular communication, playing a fundamental role in maintaining tissue homeostasis and regulating complex physiological processes [9]. Under pathological conditions, particularly in cancer, these vesicles take center stage in remodeling the tumor microenvironment, immune evasion, promoting angiogenesis, and forming the pre-metastatic niche [10]. EVs are capable of modulating the behavior of target cells by transferring different biomolecules, including proteins, lipids, carbohydrates, and nucleic acids, faithfully reflecting the functional state of the cell of origin [11]. This ability to transport specific molecular signatures gives EVs high potential as circulating biomarkers and as strategic tools for the diagnosis, prognosis, and monitoring of tumor progression, in both human and veterinary oncology.

The term ‘EVs’ refers to particles that are released from cells, delimited by a lipid bilayer, and incapable of autonomous replication, with the absence of a nucleus, encompassing a wide range of sizes on the nano- to micrometric scale [12]. The term ‘exosome’ is traditionally used to describe EVs originating from intracellular compartments, released after the fusion of multivesicular bodies (MVBs) with the plasma membrane. The process begins in the endosomal system; early endosomes mature into late endosomes or multivesicular bodies, and during this process, the endosomal membrane invaginates to generate intraluminal vesicles [13]. On the other hand, ‘ectosomes’ (also called microvesicles or microparticles) refer to those generated by direct budding from the cell membrane [12]. Distinct from these, apoptotic bodies are another type of EV and are produced when a cell dies by apoptosis and nuclear chromatin condensation occurs, followed by plasma membrane blebbing, progressing to the disintegration of the cellular contents into distinct membrane-bound vesicles, called apoptotic bodies [14].

**Figure 1:**
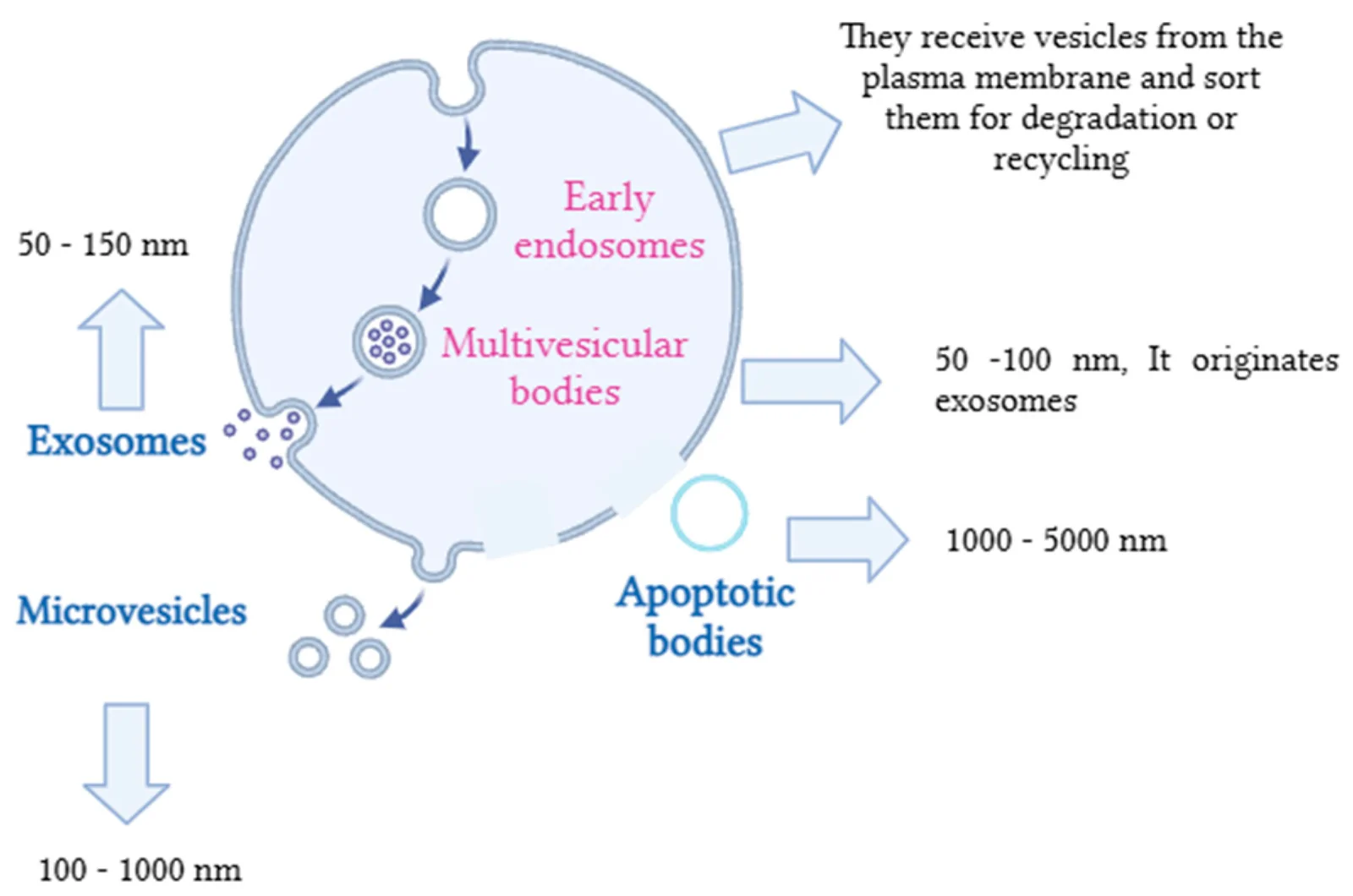
Representation of the biogenesis of EVs, as well as the size of each type. Created in BioRender. Dos Santos e Silva, L. (2026).

However, as recommended by the International Society for Extracellular Vesicles, the classification of EVs based solely on size or origin should be carried out with caution, due to the overlapping characteristics between different populations. Thus, more recent approaches suggest the use of operational criteria, such as size (e.g., small EVs < 200 nm), density, molecular composition and cellular origin, when this can be clearly demonstrated [15], as shown in Figure 1.

In cancer research, EVs are emerging as powerful tools for both diagnosis and targeted therapeutic strategies. EVs offer several advantages over other delivery systems, such as low toxicity, low immunogenicity, and high engineering capability, among other beneficial characteristics [16]. It was reported that modified EVs can deliver antitumor agents, allowing specific targeting of tumors [16,17,18,19].

To unravel the complex mechanisms of mammary tumors, this review adopts a comparative oncology approach, utilizing findings from other canine neoplasms—such as lymphomas, osteosarcomas, and melanomas, among others—as proof-of-concept models and references in understanding the conserved role of EVs.

### 1.3. Extracellular Vesicles as a Central Axis in Veterinary Oncology: Insights from Diverse Tumors for Canine Mammary Tumors

Research into EVs in veterinary oncology has evolved from experimental investigation into a strategic pillar of translational medicine. Although early studies established EVs as mediators of intercellular communication in cancer [20,21], the current literature calls for a comparative synthesis. A better understanding of data derived from various neoplasms enables the construction of a broader framework for the study of CMTs, situating them within an oncological system in which progression mechanisms mediated by EVs are frequently shared. Regarding diagnosis and systemic monitoring, the study by Andriessen et al. [22] exemplifies the importance of this translational approach: the identification of the cell-cycle regulatory protein CDC6, enriched in circulating EVs, provides a marker of aggressiveness that likely reflects the high proliferation rate observed in mammary tumors. Simultaneously, the potential of these structures to serve as liquid biopsies is supported by research on intracranial tumors [23], in which the tendency of drug-loaded EVs to target specific cell types or their selective tumor tropism suggests the possibility of an intelligent delivery system (theranostic potential).

Studies on gliomas have demonstrated that, since the degradation of miRNAs inside EVs is slower compared to that of circulating miRNAs in plasma, the assessment of miRNAs encapsulated in EVs may offer clinical advantages [24]; furthermore, although the anatomical peculiarities of the central nervous system present unique diagnostic challenges, the high specificity of miRNAs associated with EVs observed in gliomas offers a promising model. This finding reinforces the hypothesis that molecular profiling of EVs can overcome the limitations of cell-free circulating biomarkers, potentially providing a basis for identifying molecular signatures with similar discriminatory ability in CTMs.

The literature demonstrates significant alterations in the concentration and modal size of EVs between healthy and diseased dogs, with clear evidence in cases of lymphoma and adenocarcinoma. Extending this observation to CMTs is crucial, as this instability in the plasma EV profile may suggest a pathogenicity signature, reinforcing the need to integrate these physical metrics into existing molecular biomarker panels [25]. Microenvironment modulation and immune evasion represent conserved functional axes. In hematological malignancies such as lymphoma, the literature shows that EVs act not only as messengers but also as active modulators of the therapeutic response [26,27,28]. Studies indicate that lymphoma-derived EVs reduce CD8+ T-cell activity [26], interact selectively with lymphocyte subpopulations [27], and induce phenotypic changes in peripheral monocytes, thereby impacting tumor immune infiltration [29]. The mechanisms of immunosuppression and monocyte phenotypic alteration mediated by EVs, documented in lymphomas, illustrate the versatility of these vesicles as tools for tumor evasion. The ability of these vesicles to reprogram the local immune environment suggests that analogous processes may be occurring in the CMT microenvironment, warranting future investigations into how this vesicle–lymphocyte interaction contributes to malignant progression in the mammary gland. Aggressiveness and metastasis are also reflected in the molecular cargo of EVs; analysis of this cargo, including proteins and miRNAs [30,31], reveals signatures that distinguish chemotherapy-sensitive lineages from resistant ones, providing a potential model for investigating chemoresistance in CMTs. In oral melanoma, for example, the identification of RNAs [32] and long non-coding RNAs (lncRNAs) [33] correlates with metastatic status, suggesting potential biomarkers for invasion.

Similarly, in osteosarcoma, proteomic approaches identifying the PSMD14/Rpn11 protein [34] and comparative blood analyses [35] demonstrate alterations in the concentration of platelet- and leukocyte-derived EVs in diseased animals. The demonstrated ability of osteosarcoma-derived EVs to modulate T-cell proliferation and promote regulatory T-cell phenotypes offers an important parallel to breast oncology. Given that EVs from mesenchymal tumors can establish such significant immunological barriers, it is important to evaluate whether EVs derived from TMCs possess a similar capacity to orchestrate immunological tolerance, thereby facilitating tumor escape. Furthermore, the functional capacity of these vesicles to influence apoptosis [36] and modulate the regulatory T-cell phenotype [37] reinforces the fact that EVs derived from tumors of mesenchymal origin impose immunological barriers that are of potential utility in treatment strategies for CMTs.

The diagnostic potential of EVs extends to easily accessible fluids such as urine, where redox ratios and miRNA profiles (miR-182, miR-221/222) associated with urothelial carcinoma [38,39] point to specific signaling pathways (ErbB/MAPK and AKT-mTOR-S6K); it is important to investigate whether similar associations exist regarding the progression of CTMs. The use of urine as an EV source in urothelial carcinoma, combined with the correlation between metabolic signatures (redox ratio) and oncogene expression, sets a valuable precedent for research into mammary oncology. The potential of these non-invasive approaches to map signaling pathways like AKT-mTOR (known to influence chemosensitivity) highlights the need to translate such liquid biopsy strategies into clinical diagnosis and monitoring for mammary tumors. This approach is supported by studies on prostate tumors [40,41], which show how the epithelial–mesenchymal transition alters vesicular content, and on mast cell tumors [42,43], where EV overexpression of miR-21-5p is linked to lymph node metastasis. The use of EVs to monitor lymph node metastasis in mast cell tumors, particularly through the identification of miR-21-5p, marks a significant milestone in the practical application of EVs as prognostic tools. This diagnostic correlation exemplifies how insights gained from neoplasms with validated EV signatures offer a pathway to improve staging and the prediction of metastasis in mammary tumors.

In summary, the evidence accumulated across such diverse models reinforces the perspective that EVs act as architects of tumor progression. These data do not represent isolated findings but rather a convergent network: the immune escape pathways, chemoresistance markers, and metastatic signatures observed in these various neoplasms provide highly useful tools for biomarker validation and the development of more effective therapeutic strategies for CTMs.

#### EVs and Canine Mammary Tumors

In recent years, research into EVs in CMTs has advanced significantly, establishing these structures as mediators of cellular communication and promising sources of biomarkers. Recent studies demonstrate that EVs carry molecular signatures that faithfully reflect the pathophysiological state of the tumor. For instance, Gutierrez-Riquelme et al. [44] identified distinct proteomic profiles among normal tissue, adenomas, and carcinomas, highlighting biglycan (BGN) as a potential biomarker of progression. Consistently, transcriptomic analyses [45] and miRNA studies [46] confirm that vesicular content, including molecules such as miR-18a, miR-19a, and miR-181a, is modulated by the tumor phenotype. Furthermore, comparisons between primary and metastatic tumors [47] revealed an enrichment of glycolytic enzymes in EVs, highlighting the tumor’s metabolic adaptations, while Sousa et al. [48] observed variations in the abundance of proteins like keratin 18 according to histological grade. Functionally, the relevance of these structures was validated by Lee et al. [49], whose natural killer cell-derived EVs demonstrated antitumor activity, and by our group [50], which identified α-enolase as an overexpressed protein in plasma EVs from female dogs with malignant CMTs.

A combined analysis of these studies reveals a solid body of evidence but also highlights gaps that should guide future research. There is a clear consensus in the literature that EVs function as a dynamic mirror of the tumor microenvironment. Studies converge in demonstrating that, regardless of the methodology, the molecular cargo (proteins and nucleic acids) of EVs is differentially expressed according to tumor malignancy and aggressiveness. Altered metabolism, involving glycolysis and proliferative pathways, emerges as a ubiquitous signature across different approaches [45,47,50]. One point of divergence lies in quantitative parameters. While some studies observed changes in EV concentration between healthy and diseased animals [45], others, such as Sousa et al. [48], found no significant differences in total concentration, suggesting that qualitative cargo (proteins/miRNAs) may be a more sensitive indicator than the absolute number of vesicles. These discrepancies likely stem from clinical heterogeneity and, primarily, from methodological limitations regarding isolation [51,52]. As demonstrated by Moccia et al. [52], the isolation technique (ultracentrifugation versus chromatography) alters not only the yield but also the biological functionality of the sample, complicating direct comparisons between different studies.

The main current limitation is the lack of rigorous standardization in isolation and characterization protocols. High variability among study populations (different breeds, ages, and stages of mammary tumors) introduces noise that can mask specific molecular signatures. Therefore, priorities for future research include: standardization, specifically the adoption of international guidelines (such as those from the ISEV) to ensure study comparability; functional validation, moving beyond mere biomarker observation to “loss-of-function” or “gain-of-function” studies (like the work of Lee et al. [49]) to determine whether EVs are merely markers or causal agents in mammary metastasis; and placing greater emphasis on patient follow-up from diagnosis through therapeutic response, thereby establishing EVs as real-time monitoring tools (liquid biopsy).

Although EVs have demonstrated undeniable potential in the oncology of mammary tumors, further evolution of experimental methods is required to gain more relevant insights into the role of EVs in these tumors.

## 2. Integrative Analysis: The Molecular Profile of Extracellular Vesicles in Canine Mammary Tumors

To reinforce our review, we conducted an integrative analysis using CMT datasets (The full values, as well as the proteins, are described in the Supplementary Material). Klopfleisch et al. [53] performed a transcriptomic analysis of metastatic canine epithelial–stromal mammary carcinomas compared to paired healthy mammary gland tissues (*n* = 13), using the Affymetrix GeneChip Canine Genome 2.0 Array platform (GEO accession: GSE22516). Data were processed using Partek Genomic Suite Software (Version 6.4, Partek), identifying differentially expressed genes via ANOVA with FDR correction (Q < 0.001; fold change > 2.0). Additionally, Santos et al. [54] used RNA-Seq to characterize spontaneous canine ductal mammary carcinoma (T1N0M0; n = 6), comparing tumor tissue with adjacent healthy tissue. cDNA libraries were sequenced on the Illumina platform, and genes were selected based on *p* < 0.05 and |log_2_ fold change| > 1.

**Figure 2:**
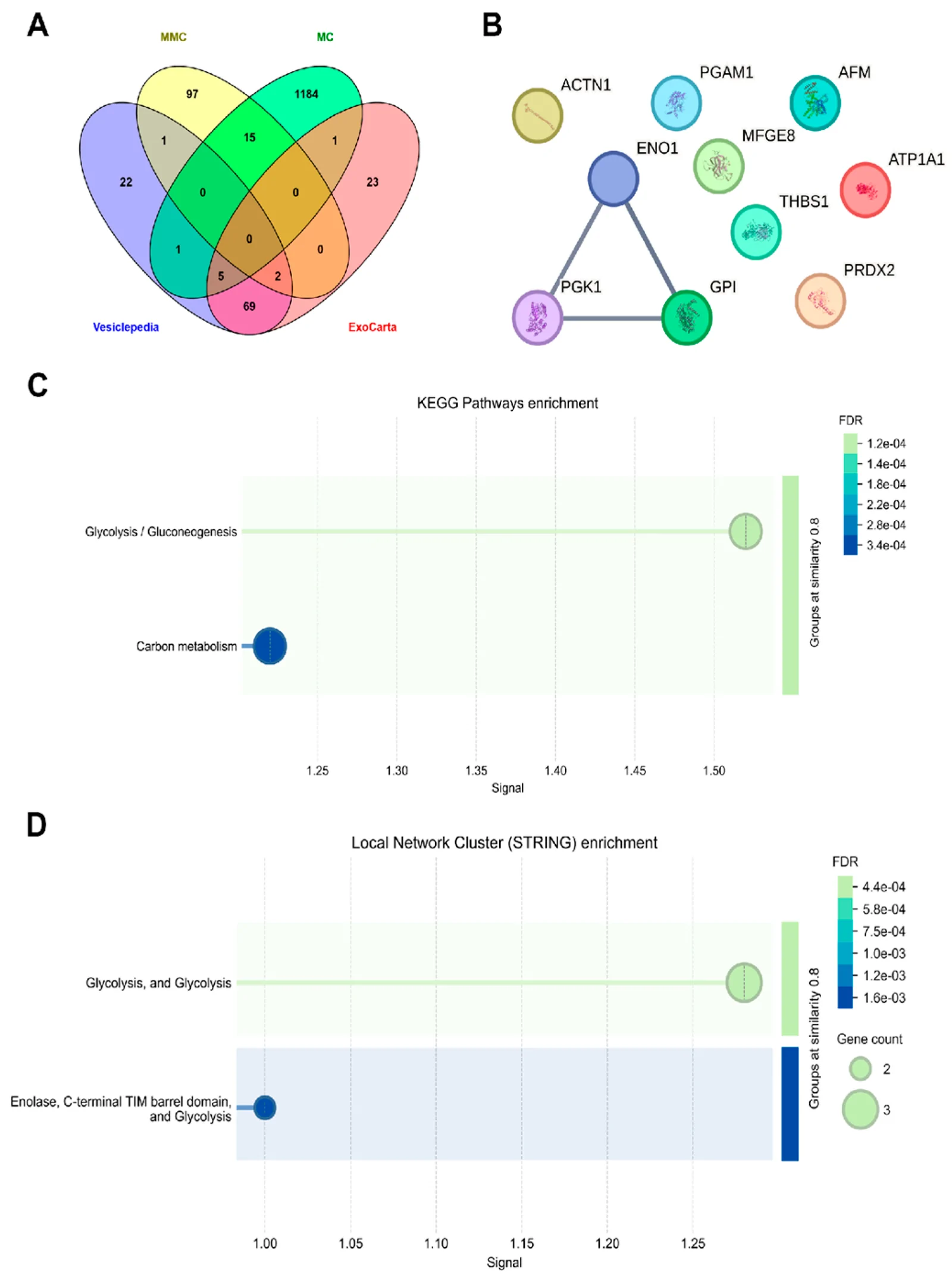
Integrative analysis of differentially expressed genes and enriched pathways associated with CMTs. (**A**) Venn diagram showing the overlap of DEG proteins among the experimental groups analyzed. Shared and exclusive proteins identified in each comparison are represented in the intersecting and non-intersecting regions, respectively. (**B**) Protein–protein interaction (PPI) network highlighting the functional interactions among the identified targets. Node represent proteins and edges represent predicted functional associations with a confidence score of 0.9. (**C**) KEGG pathway enrichment analysis demonstrating significantly enriched pathways associated with the DEGs. The x-axis represents the rich factor, while bubble size indicates the number of proteins associated with each pathway, and bubble color intensity corresponds to the false discovery rate (FDR). The connecting lines link each bubble to its corresponding pathway, and the vertical color bar indicates pathway grouping based on a similarity threshold of 0.8. (**D**) STRING Local Network Cluster enrichment analysis of biological processes related to the identified molecules. The x-axis represents the significance score, whereas bubble size and color correspond to gene count and FDR values, respectively. Connecting lines associate bubbles with the corresponding enriched terms, and the vertical color bar indicates clusters grouped according to a similarity threshold of 0.8. Darker colors indicate higher FDR values and lighter colors indicate lower FDR values.

These approaches enabled the identification of EV-associated molecules involved in tumor progression. Overlap analysis (Figure 2A) between metastatic mammary carcinoma (MMC) and non-metastatic mammary carcinoma (MC) revealed 15 common targets: Spp2, Rbp7, Itih5, Hepacam2, Efemp1, Bche, Prlr, Akap12, Nostrin, Klhl13, Txnip, Klf11, Slc2a1, Slc16a3, and Ca9. These findings suggest that this subset of EV-associated molecules represents conserved mediators in CMT biology. By cross-referencing these data with Vesiclepedia and ExoCarta, we identified: Thbs1 (common to MC and ExoCarta); Pgam1 (Vesiclepedia and MMC); Alb (Vesiclepedia and MC); Eno1 and Pgk1 (Vesiclepedia, MMC, and ExoCarta); and, finally, Atp1a1, Prdx2, Actn1, Mfge8, and Gpi (common to Vesiclepedia, MC, and ExoCarta).

To explore biological interactions, we constructed a PPI network (STRING, confidence 0.7; Figure 2B), which highlighted Eno1 as a central hub. This finding is particularly relevant, given that previous studies from our laboratory [50] had already identified the overexpression of α-enolase in plasma EVs from female dogs with malignant mammary tumors. The presence of Eno1 in the network, interacting with Pgk1 and Gpi, points to the existence of a functional glycolytic module.

Critical analysis of these findings indicates that VEs are not merely passive messengers but carriers of a metabolic program associated with aggressiveness. KEGG enrichment analysis (Figure 2C) and local clustering (Figure 2D) confirm that proteins such as Thbs1, Mfge8, Actn1, Prdx2, Atp1a1, Afm, and Pgam1 act synergistically with central metabolism. While ENO1 and Pgam1 optimize energy production via glycolysis, proteins like Thbs1 modulate the microenvironment (angiogenesis/matrix) and Prdx2 manages oxidative stress.

This convergence of evidence leads to the conclusion that the signature is consistent: the overlap between transcriptomic data [53,54] and the protein content of VEs (Vesiclepedia/ExoCarta) suggests that metabolic reprogramming, reflected by the increase in glycolytic enzymes, is a structural characteristic of CTMs. Experimental validation was achieved, as the corroboration of ENO1, both in silico and in our laboratory studies [50], positions this molecule not merely as a biomarker but as a critical component of the metastatic phenotype; there are therapeutic implications, given that the enrichment of glycolysis/gluconeogenesis pathways (low FDR) suggests that disrupting this network, or selectively inhibiting ENO1 associated with EVs, could represent a promising therapeutic strategy to curb tumor progression.

In summary, the data reinforce that the metabolic remodeling observed in EVs faithfully reflects the aggressiveness of CMTs. Network interactions centered on ENO1 and its partners (Pgk1, Gpi, and Mfge8, among others) constitute biological tools that confer adaptive advantages to the tumor, thereby making them important targets for the development of new diagnostic and therapeutic approaches in veterinary medicine.

**Figure 3:**
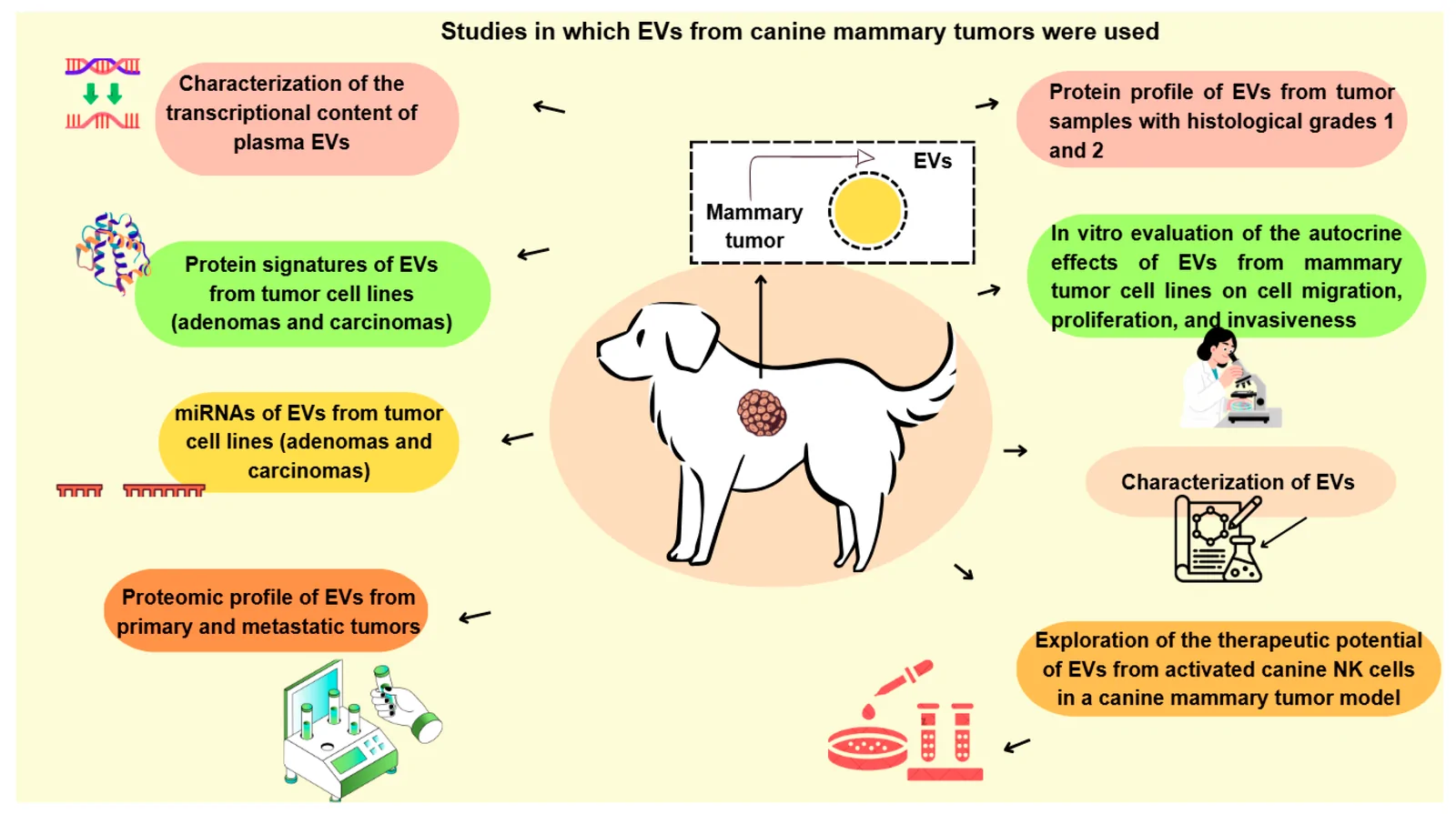
Summary of studies on EVs in CMTs: Transcriptional, protein, and miRNA characterizations of plasma-derived EVs, tumor cell lines (adenoma, adenocarcinoma, and carcinoma), and tumors with histological grades I and II were performed. Functional studies evaluated the autocrine effect of EVs on cell migration, proliferation, and invasion, in addition to investigating the therapeutic potential and the ability of these vesicles to activate natural killer cells in a mammary carcinoma model. Reference: authors’ own work.

The field of EVs applied to CMTs is expanding, as demonstrated in the present study (see Table 1 and Figure 2 and Figure 3). However, methodological advances, such as the use of small sample volumes for EV isolation [55], have facilitated EVs clinical application, expanding the potential of these vesicles in the diagnosis, prognosis, and treatment of CMTs.

## 3. Methodology

The bibliographic search strategy was designed to cover the breadth of scientific literature on EVs and canine mammary neoplasms, ensuring the comprehensiveness required for this review. A search for studies specifically addressing EVs derived from canine mammary tumor cells was conducted in the PubMed database without time restrictions, although the identified studies spanned the period from May 2018 to October 2024.

To identify relevant studies, the following search terms were used (combined via Boolean operators): “exosomes and canine mammary tumors”; “extracellular vesicle and canine mammary gland neoplasm”; and “extracellular vesicle and canine mammary tumors”. The initial search yielded a total of 31 publications. After removing duplicates and performing an initial screening based on titles and abstracts, studies that did not specifically address EVs or that referred to non-canine species were excluded. Nine articles were selected for full-text reading based on the following criteria: inclusion criteria—original studies published in journals that described isolation and molecular characterization in female dogs with mammary tumors; exclusion criteria—conference abstracts, studies on other species, or articles that did not clearly describe vesicle isolation methods, thereby compromising reproducibility.

The selected studies were then synthesized and organized to compare methodologies and consolidate available scientific evidence regarding EV biology in these tumors. This methodological approach adhered to transparency guidelines for narrative reviews, enabling the identification of both consensus findings and methodological discrepancies within the current literature. The integrative analysis aimed to validate the clinical relevance of molecular targets identified in transcriptomic studies by comparing them with the protein content of EVs documented in databases. To this end, two robust datasets were selected: one focusing on metastatic epithelial–stromal carcinomas (GEO: GSE22516, Klopfleisch et al. [53]) and the other on early-stage primary ductal carcinoma (Santos et al. [54]). The selection of these datasets was based on the complementarity between primary and metastatic tissues. The data were cross-referenced with the top 100 EV-associated molecules from the Vesiclepedia and ExoCarta databases. Protein–protein interactions (PPIs) were mapped using the STRING database (confidence > 0.7), and functional significance was determined through KEGG enrichment analysis, enabling the identification of shared glycolytic and structural modules.

For the other canine neoplasms addressed (lymphoma, osteosarcoma, urothelial carcinoma, etc.), a narrative and exploratory approach was adopted. This strategy was chosen to identify comparative oncology models illustrating specific biological mechanisms (e.g., immune evasion or metabolic reprogramming). As the objective of this section was not to provide an exhaustive account of each tumor type, but rather to contextualize the role of EVs in veterinary oncology, a targeted and representative selection of the literature was made, focusing on seminal studies and recent discoveries that serve as translational models for our central discussion on mammary tumors.

Despite growing interest in the role of EVs in oncology, this review highlights that studies specifically focused on CMTs remain scarce.

Regarding the descriptors, nine studies directly related to the topic were identified, with the term “exosomes and canine mammary tumors” proving the most effective for retrieving relevant papers. Other descriptors used, such as “extracellular vesicles and mammary gland neoplasms in female dogs” and “extracellular vesicles and canine mammary tumors”, yielded few results or merely repeated references already identified. This limitation reflects not only the scarcity of studies but also a lack of terminological standardization in the literature. Furthermore, although research remains limited regarding CMTs specifically, this review identified studies that have already explored the use of EVs in other canine neoplasms. These findings indicate that the application of EVs in veterinary oncology is expanding and could, in the future, be adapted and further developed for CMTs.

Collectively, these studies demonstrate that extracellular vesicles in CMTs act not only as promising biomarkers but also as functional elements in tumor progression and microenvironment modulation, establishing themselves as an emerging field with significant translational potential. Thus, key prospects include expanding the number of studies focused on extracellular vesicles in CMTs, as well as standardizing methodologies and descriptors. Overcoming these limitations will be crucial to consolidating the role of EVs as useful tools for both the diagnosis and treatment of CMTs.

## 4. Challenges to Be Overcome for the Clinical Applicability of Extracellular Vesicles in Veterinary Practice

The clinical applicability of EVs in veterinary oncology lies fundamentally in the concept of the liquid biopsy. Unlike conventional tissue biopsy, an invasive procedure that often requires anesthesia and captures only a spatially limited fragment of the tumor, plasma EVs offer a comprehensive view of the neoplasm’s molecular heterogeneity. In clinical practice, the use of EVs could revolutionize the monitoring of patients with mammary tumors, enabling early detection of recurrence or real-time assessment of chemotherapy response without the need for repeated surgical interventions.

However, implementing these technologies in daily practice faces significant challenges that limit their immediate translation, such as cost and infrastructure, given that the gold-standard techniques for EV isolation and characterization, such as ultracentrifugation, size-exclusion chromatography (SEC), transmission electron microscopy and nanoparticle tracking analysis, for example, require expensive equipment and specialized personnel, making them currently unfeasible for routine veterinary diagnostic laboratories. Another challenge is pre-analytical variables, since the success of the analysis depends critically on factors such as the collection tube type, centrifugation speed for plasma separation, and sample freezing time. Minor variations in these pre-analytical protocols can lead to discrepant results, hindering the reproducibility of clinical data. Another issue concerns the heterogeneity of canine patients; the vast diversity of breeds, ages, and health conditions introduces a much higher level of biological noise than is found in experimental models. Therefore, the transition to clinical application will require the development of robust biomarker panels, rather than the analysis of a single molecule, capable of distinguishing the tumor signature from metabolic changes resulting from other comorbidities common in the canine population.

## 5. Conclusions

This review demonstrated that EVs act as messengers in canine oncology, including in CMTs; despite the distinct histogenesis of canine neoplasms, comparative analysis of EVs suggests certain apparently conserved functions, such as diagnostic utility in liquid biopsies, a significant role in immune evasion via lymphocyte and monocyte modulation, involvement in glycolytic reprogramming, the transport of molecular signatures linked to metastatic potential, and participation in tumor microenvironment remodeling. These characteristics demonstrate that EVs are not merely cellular byproducts but rather strategic tools for the survival of neoplastic cells. Synthesizing these findings, it is evident that mechanisms of aggressiveness reported in other neoplasms provide a basis for investigating how CMTs utilize these same pathways to drive tumor progression.

A synthesis of data on various neoplasms suggests that mammary tumors may also share pathways of metabolic reprogramming and immune escape. Therefore, future biomarker validation in CMTs could be grounded in this comparative approach, enabling translational veterinary medicine to leverage knowledge gained from other tumors to optimize the diagnosis and treatment of dogs with mammary tumors.

## Figures and Tables

**Table 1:** Studies whose subject was EVs from dogs with mammary tumors.

Origin of EVs	Tumor Classification and/or Staging	Those Responsible for the Study
Whole-cell protein lysates (WCLs) and EV-lysates were obtained from five canine mammary cell lines—MTH53A (non-neoplastic); ZMTH3 (adenoma); MTH52C (simple carcinoma); and 1305 and DT1406TB (complex carcinoma)—and their proteins were identified by LC-MS/MS analysis	Simple and complex adenomas and carcinomas	Gutierrez-Riquelme et al. (2024) [44]
Blood plasma from dogs with mammary neoplasms	No specifications	Chen et al. (2023) [45]
Normal cultures of canine mammary epithelial cells (CMECs) and CMT cell lines	No specifications	Fish et al. (2018) [46]
Primary tumors of the canine mammary glands and metastases	No specifications	Kim et al. (2024) [47]
Plasma	Grade I, II, and III tumors Carcinoma in mixed tumor—ductal ectasia; tubular carcinoma; complex carcinoma; benign mixed carcinoma; complex adenoma; complex carcinoma; lobular hyperplasia; lipid-rich carcinoma; intraductal tubulopapillary carcinoma; micropapillary carcinoma; complex adenoma associated with mastitis; comedocarcinoma; solid carcinoma; mixed and solid carcinoma; adenoma associated with epitheliosis and squamous metaplasia and areas of solid carcinoma; osteosarcoma	Souza et al. (2024) [48]
Derivatives of canine NK cells obtained from canine peripheral blood mononuclear cells and subsequently used to treat induced mammary tumors in a canine model	No specifications	(Lee et al. 2021) [49]
Plasma from female dogs with malignant and benign mammary neoplasms	Simple carcinomas and benign mixed tumor	Novais AA et al. (2024) [50]
CMT cell line (CYPp)	No specifications	Sammarco et al. (2018) [51]
CMT cell line (CIPp)	No specifications	Moccia et al. (2023) [52]

## Data Availability

No new data were created or analyzed in this study. Data sharing is not applicable to this article.

## Supplementary Materials

The following supporting information can be downloaded at: https://www.mdpi.com/article/10.3390/ani16162478/s1.
