# Supplementary material for: Extracellular Vesicles as Emerging Tools in Canine Mammary Tumors: Potential Applications and Perspectives
Source: Animals (Basel). 2026 Aug 10;16(16):2478. doi: 10.3390/ani16162478 (PMC13508895; doi:10.3390/ani16162478)
Supplement: Supplementary file 1 [file animals-16-02478-s001.zip › animals-4447801-supplementary.pdf]

	A	B	C	D	E	F	G	H	I	J	K
1	4 grupos										
2											
3	1 common element in "MC" and "ExoCarta":						1 common element in "Vesiclepedia" and "MC":				
4	THBS1						ALB				
5											
6	1 common element in "Vesiclepedia" and "MMC":										
7	PGAM1										
8											
9	5 common elements in "Vesiclepedia", "MC" and "ExoCarta":										
10	ATP1A1										
11	PRDX2										
12	ACTN1										
13	MFGE8										
14	GPI										
15											
16	2 common elements in "Vesiclepedia", "MMC" and "ExoCarta":										
17	ENO1										
18	PGK1										
